# Metabolomic Analysis of Anti-Hypoxia and Anti-anxiety Effects of Fu Fang Jin Jing Oral Liquid

**DOI:** 10.1371/journal.pone.0078281

**Published:** 2013-10-18

**Authors:** Xia Liu, Wei Zhu, Shuhong Guan, Ruihong Feng, Hui Zhang, Qiuhong Liu, Peng Sun, Donghai Lin, Naixia Zhang, Jun Shen

**Affiliations:** 1 Naval Medical Research Institute, Shanghai, China; 2 Department of Analytical Chemistry, Shanghai Institute of Materia Medica, Chinese Academy of Sciences, Shanghai, China; 3 National Engineering Laboratory for TCM Standardization Technology, Shanghai Institute of Materia Medica, Chinese Academy of Sciences, Shanghai, China; 4 The Key Laboratory for Chemical Biology of Fujian Province, College of Chemistry and Chemical Engineering, Xiamen University, Xiamen, China; University of North Carolina at Chapel Hill, United States of America

## Abstract

**Background:**

Herba Rhodiolae is a traditional Chinese medicine used by the Tibetan people for treating hypoxia related diseases such as anxiety. Based on the previous work, we developed and patented an anti-anxiety herbal formula Fu Fang Jin Jing Oral Liquid (FJJOL) with Herba Rhodiolae as a chief ingredient. In this study, the anti-hypoxia and anti-anxiety effects of FJJOL in a high altitude forced-swimming mouse model with anxiety symptoms will be elucidated by NMR-based metabolomics.

**Methods:**

In our experiments, the mice were divided randomly into four groups as flatland group, high altitude saline-treated group, high altitude FJJOL-treated group, and high altitude diazepam-treated group. To cause anxiety effects and hypoxic defects, a combination use of oxygen level decreasing (hypobaric cabin) and oxygen consumption increasing (exhaustive swimming) were applied to mice. After a three-day experimental handling, aqueous metabolites of mouse brain tissues were extracted and then subjected to NMR analysis. The therapeutic effects of FJJOL on the hypobaric hypoxia mice with anxiety symptoms were verified.

**Results:**

Upon hypoxic exposure, both energy metabolism defects and disorders of functional metabolites in brain tissues of mice were observed. PCA, PLS-DA and OPLS-DA scatter plots revealed a clear group clustering for metabolic profiles in the hypoxia versus normoxia samples. After a three-day treatment with FJJOL, significant rescue effects on energy metabolism were detected, and levels of ATP, fumarate, malate and lactate in brain tissues of hypoxic mice recovered. Meanwhile, FJJOL also up-regulated the neurotransmitter GABA, and the improvement of anxiety symptoms was highly related to this effect.

**Conclusions:**

FJJOL ameliorated hypobaric hypoxia effects by regulating energy metabolism, choline metabolism, and improving the symptoms of anxiety. The anti-anxiety therapeutic effects of FJJOL were comparable to the conventional anti-anxiety drug diazepam on the hypobaric hypoxia mice. FJJOL might serve as an alternative therapy for the hypoxia and anxiety disorders.

## Introduction

Cells utilize oxygen to produce ATP, which is an energy source required to drive multiple cellular processes such as biosynthesis and locomotion. Maintaining oxygen homeostasis is crucial for survival and proper function of cells and organisms [1,2]. Reduced oxygen levels (hypoxia) initiate physiological changes in cells to adapt to the hypoxic environment [3], and the failure of this process results in cell death and organ dysfunction [4,5]. It has been well documented that multiple organ systems are highly affected by hypoxia, particularly the brain [6,7]. Molecular responses to hypoxia play a causal role in multiple human diseases such as cancer, stroke, pulmonary edema, inflammation, and nervous system diseases (schizophrenia, anxiety, depression etc.) [2,8,9]. Targeting hypoxia as a therapeutic strategy is a competitive choice for the treatments of the diseases mentioned above. 

Besides conventional medicines[10], traditional Chinese medicines (TCMs) such as Herba Rhodiolae (from *Rhodiola kirilowii*), St. John's Wort (from *Hypericum perforatum*), Nelumbinis Semen (from *Nelumbo nucifera*.), Tall *Gastrodia* (from *Gastrodiaelata elata*), and Ginkgo (from *Ginkgo biloba*) also have anti-hypoxia functions [11–14], and these TCMs could potentially be used for treating hypoxia related diseases. In fact, Herba Rhodiolae, which has long been used by the Tibetan people as a powerful medicinal agent to counter the high altitude hypobaric hypoxia [15,16], has been reported to show anti-depression effects [17]. Based on the knowledge including those mentioned above, we developed and patented an anti-anxiety herbal formula Fu Fang Jin Jing oral liquid (FJJOL) with Herba Rhodiolae as the major functional component, and in order to promote FJJOL for clinical use, its therapeutic mechanism should be further elucidated. 

Different from the conventional medicines, TCM single herbs and/or formulas such as FJJOL are composed of complicated chemical components which work as a holistic system in the treatment of disease. Systematic approaches such as metabolomics are therefore needed to elucidate the therapeutic mechanisms of the TCMs. As one systematic approach, metabolomics reveals whole metabolic profile changes of living systems in response to external stimuli such as hypoxia-induced injury, drug treatments [18,19]. This technology has shown value in the evaluation of the therapeutic effects and elucidation of the therapeutic mechanisms of TCMs [20,21]. In this paper, an NMR-based metabolomic study was applied to investigate the therapeutic effects of FJJOL on the high altitude forced-swimming mice model. In our experiments, the mice were divided randomly into four groups: the flatland group, the high altitude saline-treated group, the high altitude FJJOL-treated group, and the high altitude diazepam-treated group. To cause anxiety effects and hypoxic defects, a combination use of oxygen level decreasing (hypobaric cabin) and oxygen consumption increasing (exhaustive swimming) were applied to mice in the high altitude groups. Meanwhile, during the hypoxia exposure period, these mice were differentially administrated with saline, FJJOL and diazepam, respectively. After a three-day experimental handling, metabolites of mouse brain tissues were extracted by using the conventional CHCl_3_/CH_3_OH/H_2_O comprehensive extraction method. The aqueous extracts were then subjected to NMR measurements. Multivariate data analysis of PCA, PLS-DA and OPLS-DA methods were applied to analyze the NMR data and thus unravel possible correlations between the metabolite profile changes and the variations in biological pathways. The potential biomedical mechanism of FJJOL against hypoxia and anxiety was finally elucidated based on the multivariate data analysis results. 

## Experimental

### Ethics Statement

The animal works and experiment protocols were approved by the Institutional Animal Care and Use Committee of Naval Medical Research Institute.

### Reagents and materials

The conventional anti-anxiety drug diazepam, which worked as a positive control in our experiments, was purchased from Shanghai Sine Pharmaceutical Co. Ltd. All of the herbal plants, Herba Rhodiolae (Place of Origin: Tibet, China), St. John's Wort (Place of Origin: Guizhou, China), Tall *Gastrodia* (Place of Origin: Sichuan, China) and Prepared *Rehmannia* Root (Place of Origin: Henan, China) used in the present study were purchased from the Shanghai Lei Yun Shang Pharmaceutical Co. Ltd. (Shanghai, China). According to the original composition of FJJOL recorded in Chinese Patent No. ZL 2010106060, FJJOL was prepared using the following procedure. The crude drugs of Herba Rhodiolae (90 g), St. John's Wort (60 g), Tall *Gastrodia* (50 g) and Prepared *Rehmannia* Root (70 g) were pulverized and extracted in 1 L of 75% (v/v) ethyl alcohol for 12 hours at 80 °C - 90 °C. The resulting solutions were filtered and the supernatants were collected. Finally, the supernatants were condensed to a concentration of 1.0 g crude drugs/mL under vacuum. The above TCM extract was under careful quality control to ensure their identity throughout all the experiments (Figure S1 in File S1, Table S1 in File S1). The relative contents of six components present in HPLC profile (Figure S1 in File S1) were 0.96% for hypericin (1), 3.01% for gastrodin (2), 18.77% for pyrogallic acid (3), 0.45% for 5-hydroxymethyl furfural (4), 4.82% for salidroside (5), and 7.08% for tyrosol (6), respectively. 

NaH_2_PO_4_·2H_2_O and Na_2_HPO_4_·12H_2_O (all in analytical grade) were provided by Sinopharm Chemical Reagent Co. Ltd. (Shanghai, China). D_2_O (99.9% in D) was obtained from Cambridge Isotope Laboratories Inc. (Miami, FL, USA). 

### Animals experiments

Male Kunming-strain mice, 20 ± 2 g in weight, were purchased from Shanghai Experimental Animal Center of the Chinese Academy of Sciences (Shanghai, China). All animals were provided with a certified standard diet and tap water ad libitum during the experiments. They were housed on a 12/12-hour light/dark cycle in an ambient temperature of 25 ± 2 °C and 40% - 60% relative humidity.

After acclimation for seven days, the mice were divided randomly into four groups: the flatland group (n = 8, F), the high altitude saline-treated group (n = 7, HS), the high altitude FJJOL-treated group (n = 7, HF), and the high altitude diazepam-treated group (n = 7, HD). Then, all of the mice were placed individually in glass cylinders (50 cm height × 50 cm diameter) containing 30 cm depth of water at normal room temperature to practice swimming for 30 minutes a day for three days. During this period, mice in the HS group, the F group and the HF group were administrated with saline and FJJOL by intragastric at a dose of 10 g/kg•w•d, respectively. Moreover, mice in the HD group were administrated with diazepam by intragastric at a dose of 0.1 mg/kg body weight/d. After the three-day swimming training, mice of the high altitude groups were transferred to a hypobaric cabin with a simulated altitude of 5500 m and resided there for three days. To enhance hypoxic effects and cause anxiety effects, these mice were switched to a hypobaric cabin with a simulated altitude of 3500 m and forced to do exhaustive swimming three times a day. The mice in the F group were also forced to perform exhaustive swimming at the same frequency. During the exhaustive swimming period, the mice were placed individually in glass cylinders (50 cm height × 50 cm diameter) containing 30 cm depth of water at 22 ± 2 °C. After the third disappearance of their whole head under water, the mice were considered to be immobile. They were then removed from the glass cylinders and put back into the cabin with the higher simulated altitude of 5500 m. During the hypoxia exposure period, mice in high altitude groups were differentially administrated with saline, FJJOL and diazepam as described above. 

### Sample collection and NMR experiments

At the end of experiments, mice from each group were subjected to ethological study by following the protocols described in the previously published papers [22–25]. Right after the ethological experiments had been done, all of the experimental mice were sacrificed by decapitation. The brain tissue samples from the left hemispheres were then quickly removed from each mouse, snap-frozen in liquid nitrogen and subsequently stored at -80 °C before NMR analysis.

Lyophilized aqueous brain extracts were prepared using the methanol/chloroform/water system as previously described [26,27]. Frozen left brain tissues were placed in the hard tissue-homogenizing tubes (Bertin Technologies) with small ceramic beads and added in 4 mL/g (wet mass) methanol, 2.85 mL/g (wet mass) ultrapure water and 4 mL/g (wet mass) chloroform. The mixtures were allowed to thaw for 3 min and followed by 2 × 20 s beating of 5,600 rpm and 20 s pause between the bead beatings using a tissue homogenizer (precellys 24, Bertin technologies, Villeurbanne, France). After a 15-minute incubation at 4 °C, the extract samples were centrifuged at 11,000 g for 10 min at 4 °C. The solution samples were separated into an upper methanol/water phase and a lower chloroform phase. The upper aqueous phase was transferred into a marked EP tubes and lyophilized. The powder of the extract was dissolved in 600 μL of phosphate buffer (0.2 M Na_2_HPO4/0.2 M NaH_2_PO_4_, pH 7.4), vortexed and then centrifuged at 11,000 g for 10 min at 4°C. Aliquots of the supernatant (500 μL) were transferred into 5-mm NMR tubes, and then 50 μL of D_2_O was added for NMR measurements.

### NMR analysis

All ^1^H NMR spectra were acquired on a Bruker Avance Ⅲ-500 MHz (proton frequency) spectrometer equipped with a 5 mm dual ^1^H/^13^C Z-Grad CryoProbe™ (Bruker biospin, Germany), operating at 500.13 MHz for ^1^H. Solvent-suppressed 1D ^1^H NOESY spectra (NoesyPr1d) were acquired using the pulse sequence [RD-90-t_1_-90-t_m_-90-ACQ] with a mixing time (t_m_) of 100 ms. Water suppression was achieved by irradiation of the water resonance during the recycle delay (RD) of 4 s and the mixing time. The 90° pulse length was adjusted to about 10.35 μs. t_1_ was set to 4 μs. A total of 4 dummy scans and 256 free induction decays (FIDs) were collected into 120 k data points, using a spectral width of 10 kHz, giving an acquisition time (ACQ) of 6.13 s. Measurements for all samples were carried out at 25 °C.

To aid resonance assignments of 1D ^1^H NMR spectra, 2D pulsed field gradient COrrelation SpectroscopY (gCOSY), together with 2D homonuclear Total Correlation Spectroscopy (TOCSY) were acquired on selected samples. In 2D NMR experiments, 64 transients per increment and 256 increments were collected into 1024 data points, with spectral width of 8 kHz in both dimensions.

### Multivariate statistical techniques

To exploit quantitative metabolic information embedded in the spectra, the free induction decays (FIDs) of 1D ^1^H NOESY spectra were multiplied with an exponential function and line-broadening factor of a 0.3-Hz prior to Fourier transformation. The NMR spectra were manually phased, corrected for baseline distortion, referenced to the methyl group of lactate at δ 1.330 and carefully aligned using the software of MestReNova (Version 8.0, Mestrelab Research SL). The spectral region of each metabolite was integrated into one bin. The resulting 28 metabolites were normalized to the sum of the spectral intensity to compensate for differences in the concentrations of samples. Subsequently, the integral values were mean centered for PCA (Figure S3 in File S1), PLS-DA and OPLS-DA by SIMCA-P+12.0 software package (Umetrics, Umeå, Sweden). The PCA and PLS-DA score plots were visualized with the first principal component (t[1]) and the second principal component (t[2]), while OPLS-DA were visualized with the first principal component (t[1]) and the orthogonal component (to[1]). The parameters Q2 (cum) and R2X (cum) were computed to test the validity of the model against overfitting, where R2X (cum) is the total variation explained in the data and Q2 (cum) is the cross-validated explained variation with increasing reliability as Q2 (cum) approaches 1 [28]. The six-fold cross-validation method and permutation test for 500 times with the first component were carried out to measure the robustness of the model, where if Q2 (max) obtained from permutation test is less than or equal to Q2 (cum) obtained from OPLS-DA, the established OPLS-DA model is robust. The correlation coefficients of Pearson correlation between the variations and the first component of OPLS-DA were extracted from correlation-loading plots of OPLS-DA models (Figure S4 in File S1). Cutoff values with significant levels of 0.05 were used to identify key variables that were responsible for the discrimination of groups. Additionally, the relative changes of metabolites between groups were calculated using the normalized integral, i.e. (*C*_*A*_ *-C*_*B*_)*/C*_*B*_, where C_A_ and C_B_ stand for the mean metabolite integrals of two groups in the OPLS-DA models. 

### Univariate statistics of metabolites' integral

Group means of metabolites' integral are expressed as the mean ± std. Significant differences in the mean values were evaluated by Student’s *t*-test. Intergroup variation was measured by one way analysis of variance (ANOVA) followed by Bonferroni correction. Statistical significance was considered at *p* < 0.05. Statistical analyses were performed with SPSS 17.0. 

## Results

**Figure 1:**
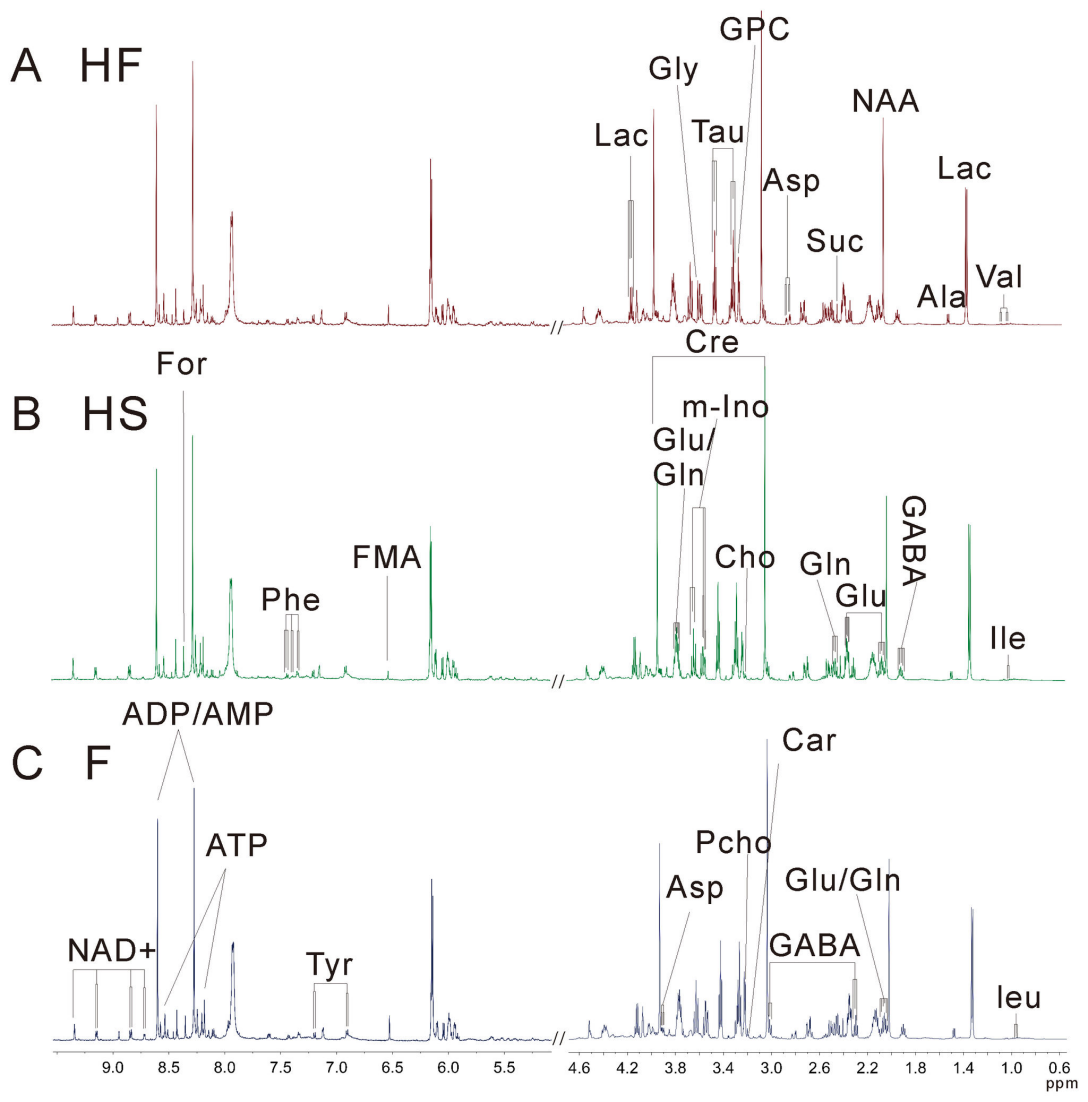
500-MHz ^1^H NMR NOESY spectra (δ 0.5-4.7, 5.0-9.6) of aqueous extracts from brain tissues of mice in groups HF (A), HS (B), and F (C). The abbreviations of metabolites were shown in Table 1.

All the mice were divided randomly into four groups: flatland group (F, n = 8), high altitude saline-treated group (HS, n = 7), high altitude TCM formula-treated group (HF, n = 7), and high altitude diazepam-treated group (HD, n = 7). After a three-day differential handling, aqueous extracts of brain tissues from all four groups were subjected to NMR metabolomic studies. A total number of 28 brain metabolites were identified by fully mapping of chemical shifts, coupling patterns and coupling constants to previously reported data (Figure 1: data for the HD group were not shown, Figure S2 in File S1) [29,30]. NMR resonance assignments of 28 identified metabolites, which include metabolites involved in glycolysis and the Krebs cycle (lactate, fumarate, malate, succinate), amino-acids (isoleucine, leucine, valine, alanine, glutamine, lysine, glutamate, aspartate, glycine, phenylglycine, tyrosine), choline metabolites (sn-glycero-3-phosphocholine, phosphoylcholine, choline), myo-inositol, taurine, γ-aminobutyric acid, isobutyrate, creatine, formate, ATP, ADP/AMP, and nicotinamide adenine dinucleotide are summarized in Table 1.

**Table 1:** Resonance assignments of 28 metabolites in ^1^H NMR spectra of aqueous extracts of brain tissues.

Metabolite (abbreviation)	Groups	δ ^1^H(ppm) in PBS buffer (pH=7.4) ^#^	
leucine(Leu)	α-CH, β-CH_2_, γ-CH, δ-CH_3_, δ-CH_3_	3.73(m), 1.73(m), 1.70(m),1.69(m), 0.97(d), 0.96(d)	
isoleucine(Ile)	α-CH,β-CH, γ-CH_3_, half γ-CH_2_, half γ-CH_2_, δ-CH_3_	3.67(d), 2.00(m), 1.01(d), 1.42 (m), 1.21(m), 0.94(t)	
valine(Val)	α-CH, β-CH, γ-CH_3_, γ-CH_3_	3.60(d), 2.26(m), 1.05(d), 0.99(d)	
isobutyrate(IB)	α-CH, 2×β-CH_3_	2.49(m), 1.06(d)	
lactate(Lac)	α-CH, β-CH_3_		4.13 (q), 1.34(d)
analine(Ala)	α-CH, β-CH_3_	3.78(q), 1.49(d)	
lysine(Lys)	α-CH, β-CH2, half γ-CH_2_, half γ-CH_2_, δ-CH_2_, ε-CH_2_	3.75(t), 1.90(m), 1.452(m), 1.50(m), 1.72(m), 3.02(t)	
N-acetylaspartate(NAA)	α-CH_2_	2.10(s)	
glutamate (Glu)	α-CH, half β-CH_2_, half β-CH_2_, half γ-CH_2_, halfγ-CH_2_	3.78(t), 2.13(m), 2.06(m), 2.34(m), 2.37(m)	
γ-aminobutyric acid (GABA)	α-CH_2_, β-CH_2_, γ-CH_2_	2.28(t), 1.89(m), 3.00(t)	
succinate(Suc)	2×CH_2_	2.41(s)	
glutamine(Gln)	α-CH, β-CH_2_, γ-CH_2_	3.78(t), 2.44(m), 2.14(m)	
malate(Mal)	α-CH, β-CH_2_	2.68(dd), 2.35(dd)	
aspartate(Asp)	α-CH, β-CH_2_	3.89(dd), 2.82(dd), 2.69(dd)	
creatine(Cr)	α-CH_2_, N-CH_3_	3.95(s), 3.04(s)	
choline(Cho)	^1^CH_2_^2^,CH_2_, N(CH_3_)_3_	4.05(t), 3.51(dd), 3.21(s)	
phosphoylcholine(PC)	^1^CH_2_^2^,CH_2_, N(CH_3_)_3_	4.18(m), 3.60(t), 3.22(s)	
sn-glycero-3-phosphocholine(GPC)	^1^ CH_2_^2^,CH_2_, N(CH_3_)_3_. glycerol: half ^1^CH_2_, half ^1^CH_2_^2^,CH, half ^3^CH_2_, half ^3^CH_2_	4.33(m), 3.68(m), 3.23(s), 3.60(dd), 3.68(dd), 3.90(m), 3.87(m), 3.94(m)	
taurine(Tau)	^1^CH_2_^2^,CH_2_	3.43(t), 3.27(t)	
myo-inositol(m-Ino)	^1^CH ^2^,CH ^3^,CH ^4^,CH ^5^,CH ^6^,CH	3.54(dd), 4.07(t), 3.54(dd), 3.63(t), 3.29(t), 3.63(t)	
glycine(Gly)	α-CH_2_	3.57(s)	
fumarate(FMA)	CH	6.52(s)	
tyrosine(Tyr)	phenyl moiety: α-CH, β-CH, half β-CH_2_, half β-CH_2_	7.19(d), 6.92(d), 3.05(dd), 3.19(dd)	
phenylalnine(Phe)	phenyl moiety:α-CH, β-CH, γ-CH, half β-CH2, half β-CH2, α-CH	7.33(d), 7.43(t), 7.37(t), 3.98(dd), 3.27(dd), 3.12(dd)	
ATP^1^	adenine moiety ^2^:CH, 8CH, NH_2_	8.58(s), 8.27(s), 6.14(d)	
ADP/AMP^2^	adenine moiety ^2^:CH, 8CH', NH_2_	8.60(s), 8.27(s), 6.14(d)	
formate(For)	CH	8.46(s)	
nicotinamide adenine dinucleotide(NAD)	Nicotinamide moiety: α-CH, α-CH, γ-CH, β-CH, NH_2_(CO). Adenine moiety ^2^:CH, 8CH, NH_2_	9.32(s), 9.13(d), 8.82(d), 8.20(m), 6.08(s) 8.41(s), 8.16(s), 6.03(d)	

^#^ s, singlet; d, doublet; t, triplet; q, quartet; m, many peaks.

^1^ ATP, adenosine triphosphate.

^2^ ADP/AMP, adenosine diphosphate/adenosine monophosphate.

### Brain defects induced by hypobaric hypoxia

**Figure 2:**
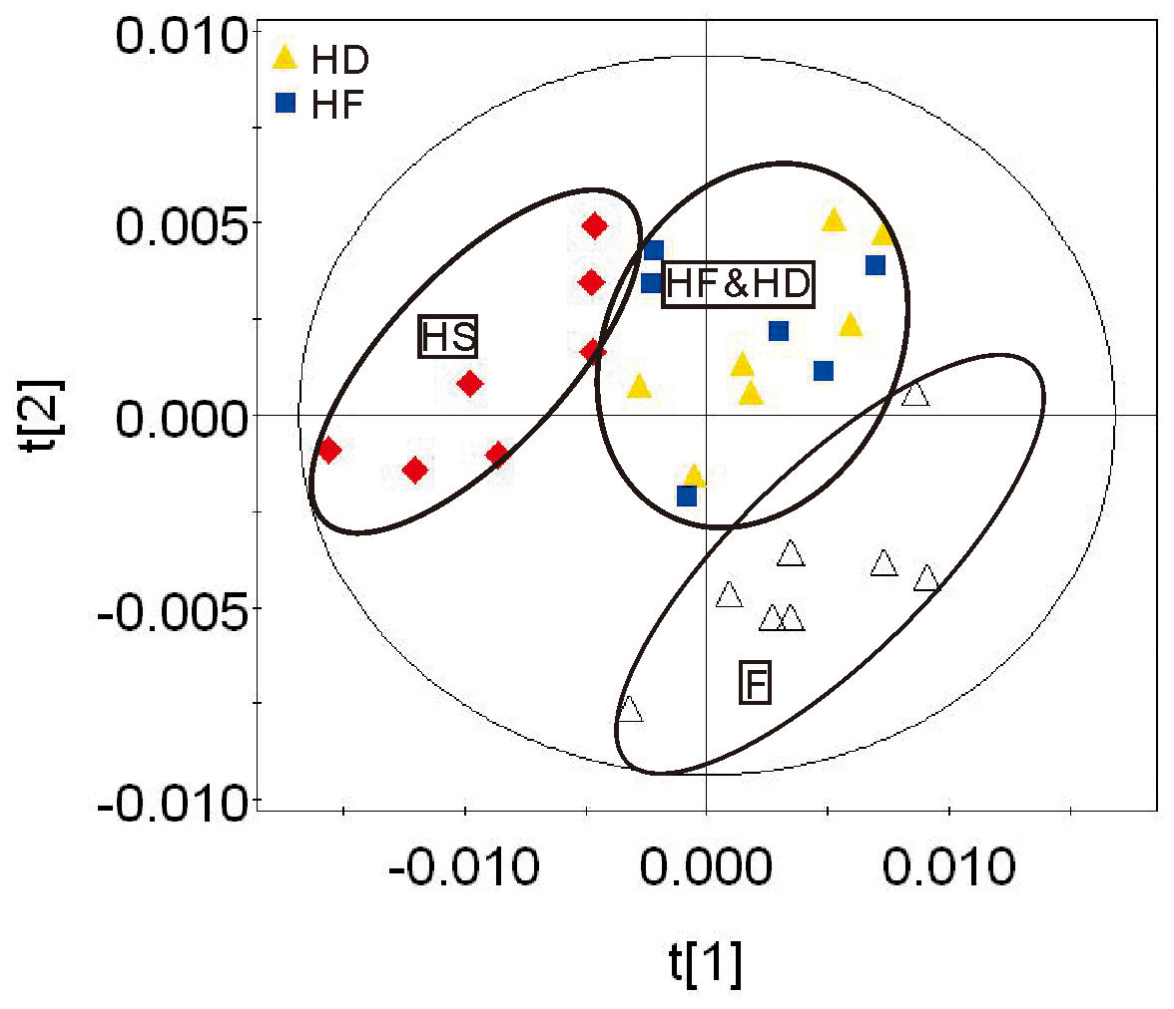
PLS-DA scatter plots of ^1^H NMR data of aqueous extracts from brain tissues of mice in groups HF, HD, HS, and F.

**Figure 3:**
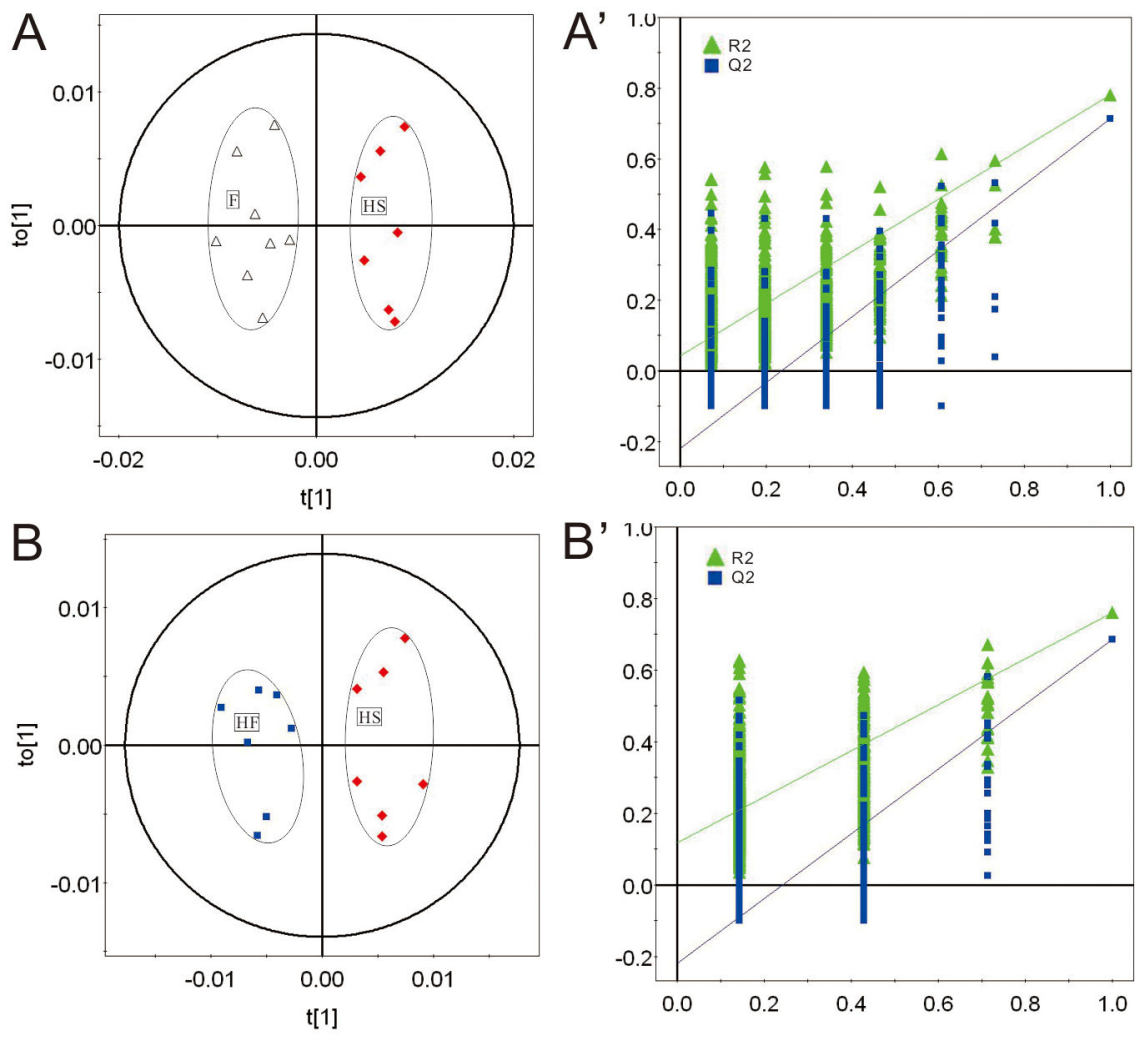
OPLS-DA scatter plots derived from ^1^H NMR spectra of aqueous extracts of mouse brain tissues and validation plots from the HS and the F groups (R2X(cum)=0.84 and Q2(cum)=0.86) (A, A' **), and the HF and the HS groups (R2X(cum)=0.64 and Q2(cum)=0.81) (B, B')**. The validation plots were obtained by using a permutation test that was randomly permuted for 500 times with the first component extracts. ▲ is for R2Y (cum), and ■ is for Q2 (cum). The vertical axis of the validation plots represented the R2 and Q2 values, and the horizontal axis (A', B') represented the correlation coefficients.

In mammalian cells glucose is the major source material for ATP production. Under normoxic conditions ATP molecules are mainly produced through metabolism of glucose, which is composed of three relay pathways: oxygen-independent pathway of glucose to pyruvate in cytoplasm, citric acid cycle (Krebs cycle, TCA cycle), and oxygen-dependent electron transfer chain in mitochondria. ATP production efficiency by metabolism of glucose under normoxia condition is efficient, and 2 and 4 ATP molecules per glucose are produced by pathway of glucose to pyruvate and TCA cycle coupled electron transfer chain, respectively. Since the TCA cycle coupled ATP production process is oxygen dependent, it is expected that reduced oxygen level (hypoxia) will significantly affect this process. Under hypoxic conditions anaerobic glycolysis begins to play a dominant role for ATP production, and pyruvate is converted into lactate in the cytoplasm instead of going into the TCA cycle in mitochondria. Hypoxic stress down-regulates oxygen-dependent glycolysis of glucose and triggers mitochondrial oxidative stress [31–33]. To determine if the hypobaric hypoxia mice model was successfully set up, relative quantitative analysis of 28 identified metabolites from brain tissues of HS and F groups was applied. Consistent with previous knowledge, hypobaric hypoxia caused significant energy metabolism defects in brain tissues of mice. Upon hypoxic exposure, ATP level was reduced, major functional components of the Krebs cycle including fumarate and malate were significantly altered, and lactate, the signature metabolite of the anaerobic glycolysis, was greatly elevated (Table 2). Besides energy metabolism defects, disorders of functional metabolites such as glycine, GABA (neurotransmitter), and NAA (neurotransmitter precursor), which indicated functional impairments in the hypoxic mouse brain, were also observed (Table 2). In fact, down-regulation of GABA caused by hypoxia pointed to an anxiety effect in mice [34]. OPLS-DA and PLS-DA scatter plots revealed a clear group clustering for metabolic profile in the normoxia versus hypoxia samples (Figure 2, Figure 3A, 3A’). 

**Table 2:** Quantitative comparison of metabolites found in aqueous extracts of brain tissues of F, HF and HS group mice.

Metabolites	Integral in F group ^a^ (mean±std)*10^-4^	Integral in HF group ^a^ (mean±std)*10^-4^	Integral in HS group ^a^ (mean±std)*10^-4^	% Average change (HF vs. HS)^b^	|r|(*p* ^d^)(HF vs. HS) ^c^ (|r|>=0.53)	% Average change (HS vs. F) ^b^	|r|(*p* ^d^)(HS vs. F) ^c^ (|r|>=0.51)
ATP	2.94±0.56	3.07±1.36	2.36±0.33	30.1	0.53(0.09)	-19.7	0.63(0.01)
Tyr	3.30±0.31	4.28±0.59	3.97±0.59	7.8	0.18(0.17)	20.3	0.67(0.01)
FMA	3.10±0.74	2.94±1.21	1.88±0.30	56.3	0.63(0.01)	-39.4	0.67(0.00)
Gly	258±26.0	279±21.5	236±19.1	18.2	0.62(0.04)	-8.5	0.84(0.00)
m-Ino	99.1±8.08	94.5±7.41	88.7±7.9	6.5	0.64(0.08)	-10.5	0.78(0.01)
GPC	201±12.3	163±14.3	146±11.6	11.6	0.59(0.01)	-27.4	0.93(0.00)
Pcho	120±11.0	103±9.00	108±8.70	-4.6	0.44(0.15)	-10.0	0.43(0.01)
Cho	27.8±4.83	31.7±22.4	22.6±1.80	40.2	0.44(0.14)	-18.7	0.56(0.01)
Cr	678±28.5	668±55.2	715±24.3	-6.6	0.43(0.14)	5.5	0.64(0.01)
GABA	145±9.17	152±11.1	138±10.7	10.1	0.64(0.01)	-4.8	0.37(0.06)
Gln	235±7.77	260±13.3	254±19.7	2.4	0.04(0.43)	8.1	0.76(0.00)
Suc	52.2±3.71	48.3±6.28	53.6±5.36	-9.9	0.61(0.05)	2.7	0.27(0.27)
Mal	90.0±7.28	95.0±5.81	99.5±3.70	-4.5	0.61(0.04)	10.6	0.84(0.00)
NAD	4.75±0.98	4.31±1.58	5.43±0.57	-20.6	0.63(0.04)	14.3	0.40(0.06)
NAA	388±8.28	406±31.0	418±13.2	-2.9	0.42(0.15)	7.7	0.81(0.00)
Lac	594±45.2	594±48.3	672±65.0	-11.6	0.72(0.01)	13.1	0.73(0.01)

^a^ The relative integrals of metabolites were determined from 1D ^1^H NMR analysis of brain aqueous extracts of each group mice.

^b^ Values are represented as the fold-induction of peak integral between groups.

^c^ The absolute values of correlation number extracted from the correlation plots of OPLS-DA models. The cutoff values are 0.53 in the correlation-loading plot of HF vs. HS (Figure S4A in File S1) and 0.51 in the correlation-loading plot of HS vs. F (Figure S4B in File S1).

^d^ The *p* values were obtained from student's *t*-test.

### Therapeutic effects of FJJOL on hypobaric hypoxia mice

*Rhodiola rosea* (Hong Jing Tian), one of the most famous anti-hypoxia TCMs, has long been used by the Tibetan people as a powerful medicinal agent to counter the high altitude hypobaric hypoxia [15,16]. The TCM formula (FJJOL) composed of *Rhodiola rosea* (the major functional component) and three other herbs had been patented with anti-anxiety effects (Chinese patent No. ZL20101060604032.4). The anti-anxiety therapeutic effects of FJJOL were unraveled by ethological data (Table S2 in File S1, Table S3 in File S1). To further elucidate the therapeutic mechanism, both relative quantitative analysis and multivariate analysis of 28 identified metabolites from brain tissues of HS, HF, HD and F groups were applied. FJJOL showed recovering effects on pathways of ATP production. Levels of ATP and metabolites such as fumarate, malate and lactate, which were perturbed by hypoxia, recovered toward normal (Figure S4 in File S1, Table 2). Moreover, disorders of functional metabolites such as glycine, GABA, and NAA were fully or partially repaired (Figure S4 in File S1, Table 2). PCA (Figure S3 in File S1), PLS-DA (Figure 2) and OPLS-DA (Figure 3) scatter plots revealed a clear group clustering for metabolic profile in the saline-treated versus drug-treated hypoxic samples, and the FJJOL-treated group and anti-anxiety drug diazepam-treated group fell into the same region (Figure S3 in File S1, Figure 2, Figure 3B, 3B’).

## Discussion

Oxygen is used by cells to produce ATP, which is the energy source used to drive multiple cellular processes. Maintaining oxygen homeostasis is crucial for survival and proper function of cells and organisms [1,2]. The mammalian brain is one of the most oxygen-sensitive organs. Reduced oxygen level (hypoxia) could cause neurologic dysfunction, and hypoxia is highly associated with the occurrence and development of multiple nervous system diseases such as schizophrenia and depression. It is known that some traditional Chinese medicines such as *Rhodiola rosea* (Hong Jing Tian) have anti-hypoxia functions [12,14,17], and these TCMs might be used in the treatment of hypoxia related diseases. The TCM formula (FJJOL) composed of *Rhodiola rosea* (the major functional component) and three other herbs had been patented with anti-anxiety effects (Chinese patent No. ZL20101060604032.4). However, the clinical practice of this formula is slowed down by the limited understanding of the therapeutic mechanism. In this paper, ^1^H NMR-based metabolomic analysis was used to elucidate the therapeutic effects of TCM formula mentioned above on the hypobaric hypoxia mice. 

**Figure 4:**
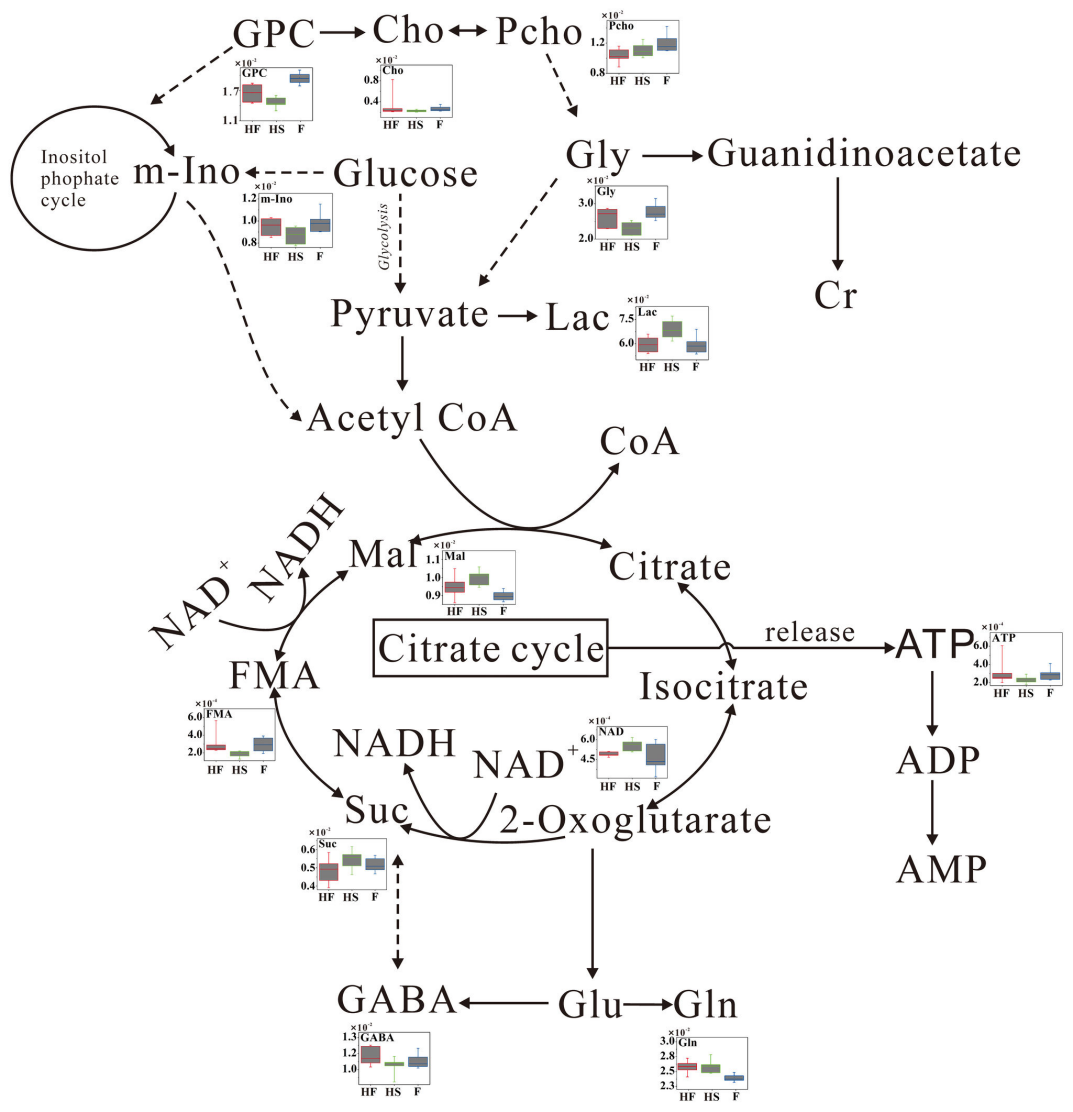
Potential metabolic pathways disturbed by hypobaric hypoxia exposure and altered by FJJOL.

Metabolomic analysis is a powerful approach to ascertain metabolic signatures from a complex combination of small molecules in biological fluid [30,35,36] and/or tissue [37–40]. In this paper, the hypobaric hypoxic mouse model with anxiety symptoms was set up by oxygen decreasing (hypobaric cabin) and oxygen consumption increasing (forced-swimming). ^1^H NMR spectroscopy and multivariate analysis were used to identify metabolic pathways affected by FJJOL. It was found that energy metabolism of brain tissues in high altitude forced-swimming mice was activated by administration of FJJOL. The levels of key metabolites in the Krebs cycle and glycolysis such as fumarate, malate, lactate and ATP, which were perturbed by the hypobaric hypoxia environment, recovered toward normal (Table 2, Figure 4). However, the level of another energy metabolism-related metabolite, creatine, which served as a reservoir for high-energy phosphates and was also disturbed by hypoxia exposure, was not recovered by the FJJOL treatment.

Other than the recovering effects on energy metabolism, FJJOL was also able to produce an anti-anxiety like effect in the high altitude forced-swimming mice. GABA is a major inhibitory neurotransmitter in brain, which is mainly synthesized in GABAergic neurons and holds a well-known anti-anxiety function [41–43]. In our work, as shown by Table 2 and Figure 4, the levels of GABA and another inhibitory neurotransmitter glycine were reduced in the hypobaric hypoxia mice and elevated in the FJJOL-treated animals. These data indicated that FJJOL might affect the functions of GABAergic and glycine receptor enriched neurons. The elevated GABA level indicated an enhancement of GABAergic neuron function, which would then have a significant impact on the function of temporal cortex to improve anxiety symptoms [44,45]. Moreover, the decreased level of NAA in the HS group was also observed, which indicated the neuronal dysfunction and the reduction of neuron density upon the hypoxia exposure. However, the FJJOL treatment showed no significant rescue effect for the disturbed NAA level.

Another interesting result in this study was that the disturbed level of GPC, which was thought to be a marker of cell density and membrane turnover, recovered toward normal in the FJJOL-treated group. It has been reported that there was a significant correlation between the anxiety symptoms and the level of GPC in brain [46]. The recovered level of GPC in the HF group might also contribute to the anti-anxiety therapeutic effects presented by the FJJOL. Besides, since GPC was reported to play a key role in maintaining the membrane integrity of the cell [47], the anti-anxiety effects of the FJJOL might partially achieved through its membrane protecting action.

In conclusion, based on ^1^H NMR spectra of brain tissues, we identified the metabolic profiles of the high altitude forced-swimming mice treated with or without FJJOL and the flatland forced-swimming mice. FJJOL ameliorated hypobaric hypoxia effects by regulating energy metabolism, inhibitory neurotransmitters metabolism, and improving the symptoms of anxiety after stress stimulation. Our work revealed the therapeutic mechanism of the patented FJJOL for the first time, which would speed up the clinical application process of the formula.

## Supporting Information

File S1 Supplementary materials.
(DOC)
