# Supplementary material for: Metabolomic Analysis of Anti-Hypoxia and Anti-anxiety Effects of Fu Fang Jin Jing Oral Liquid
Source: PLoS One. 2013 Oct 18;8(10):e78281. doi: 10.1371/journal.pone.0078281 (PMC3799728; doi:10.1371/journal.pone.0078281)
Supplement: File S1 — Supplementary materials. (DOC) [file pone.0078281.s001.doc]

**Supplementary Materials**

**Figure S1.** High performance liquid chromatography (HPLC) fingerprint of the FJJOL: hypericin (1), gastrodin(2), pyrogallic acid (3), 5-hydroxymethyl furfural (4), salidroside(5), tyrosol (6).

**Figure S2.** All collected ^1^H NMR spectra of aqueous extracts from brain tissues of mice in groups HF, HS and F.


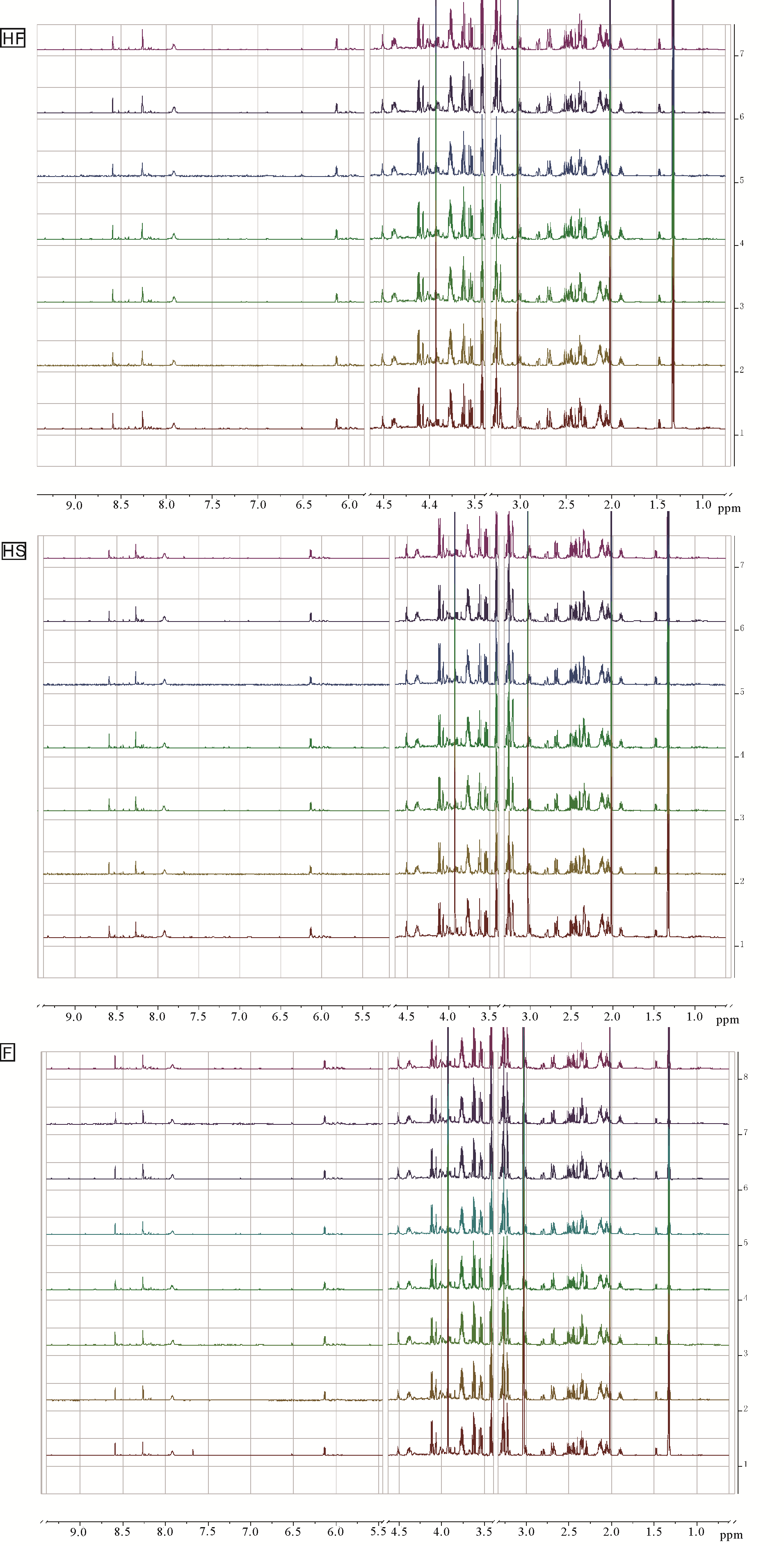


**Figure S3.** PCA scatter plots of ^1^H NMR data of aqueous extracts from brain tissues of mice in groups HF, HD, HS, and F.


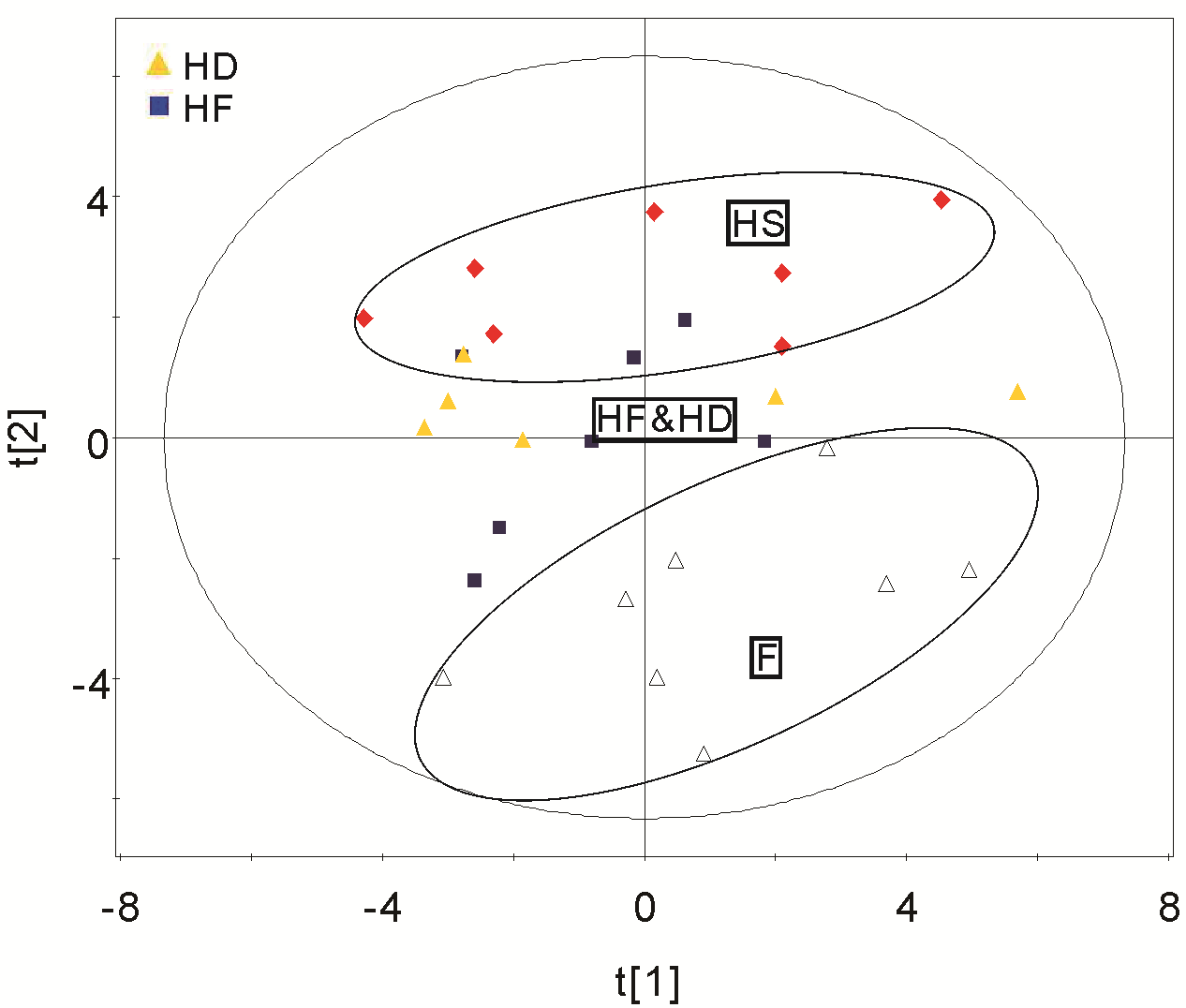


**Figure S4.** Correlation-loading plots derived from ^1^H NMR spectra of aqueous extracts of brain tissues of mice in (A) HF vs. HS and (B) HS vs. F.


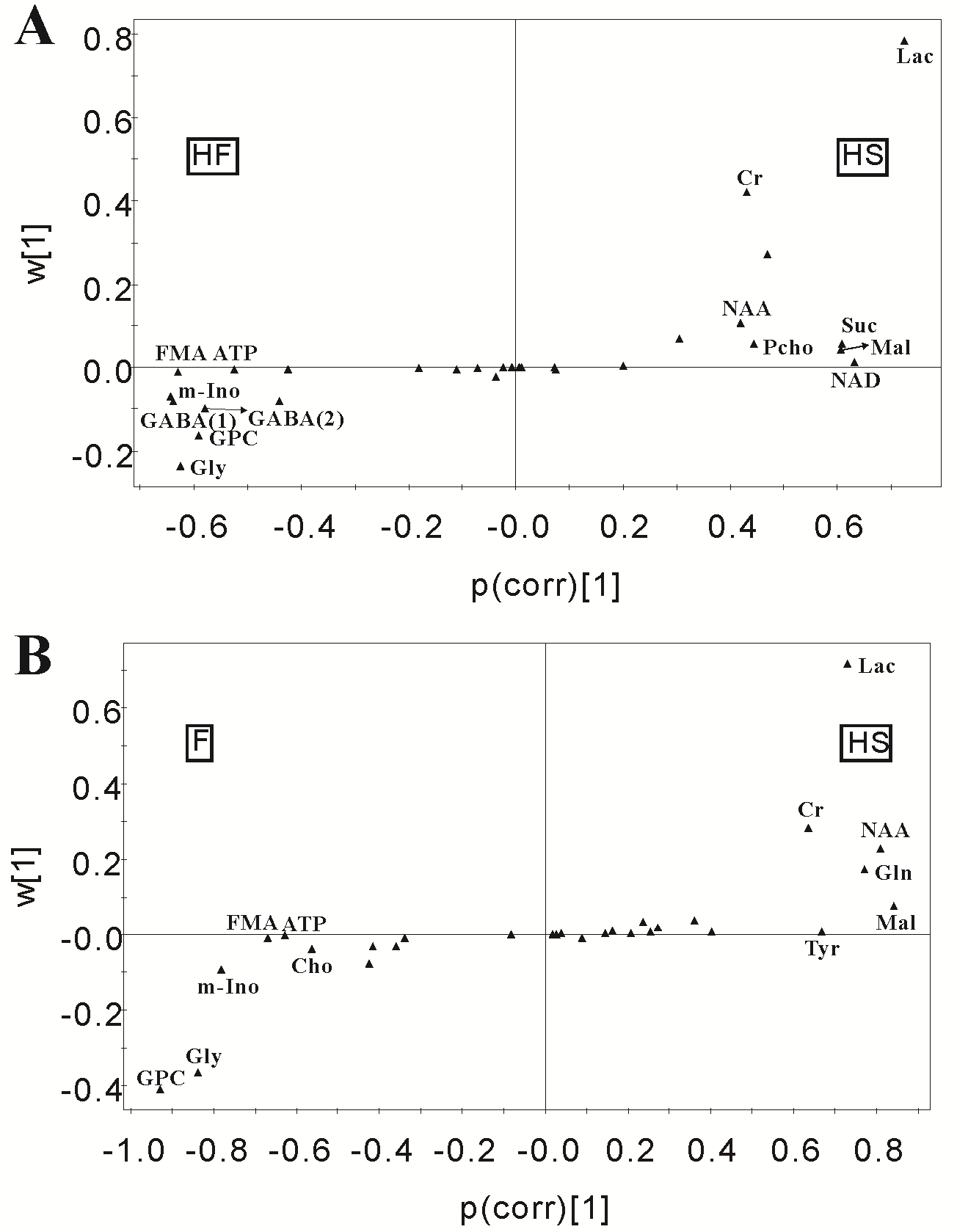


**Table S1.** The protocol used for chromatographic separation of the extract. The separation of the extract was performed on the Agilent Technology 1100 Series HPLC with a SHISEIDO CAPCELL PAK C18 column (250 mm x 4.6 mm, 5 μm) by linear gradient elution using acetonitrile-0.026% phosphoric acid solution at a flow rate of 1.2 mL/min. The elution was monitored at 230 nm and the column temperature was set at 30 °C.

| **Time (min)** | **acetonitrile (%)** | **0.026% phosphoric acid solution (%)** |
| --- | --- | --- |
| **0** | 7.5 | 92.5 |
| **17** | 10 | 90 |
| **22** | 12 | 88 |
| **30** | 17 | 83 |
| **35** | 17 | 83 |
| **40** | 30 | 70 |

**Table S2. Spontaneous behavior of mice in open field test after 3 days stimulation ($\overline{x}\pm s$).**

| **Group** | **movement distance**  **(m)** | **average moving speed**  **(mm/s)** | **resting time**  **(s)** | **central zone**  **movement distance**  **(m)** | **central zone movement time**  **(s)** | **excretion of disposal**  **(numbers)** |
| --- | --- | --- | --- | --- | --- | --- |
| **HS** | 12.12±4.91 | 19.65±7.95 | 332.60±45.69 | 1.14±0.99 | 19.82±15.11 | 4.75±1.99 |
| **HD** | 12.59±4.14 | 20.05±6.57 | 311.09±59.54 | 1.01±0.77 | 20.30±15.56 | 2.88±2.10^*^ |
| **HF** | 16.63±6.21^*^ | 29.04±8.37^*^ | 179.72±77.38^**^ | 2.45±0.97^**^ | 42.28±22.17^**^ | 2.88±1.46^**^ |
| **F** | 17.32±5.07^#^ | 30.88±6.25^#^ | 156.97±56.94^##^ | 2.23±0.87^##^ | 41.68±19.45^##^ | 2.08±1.03^##^ |

^*^*p*<0.05, ^**^*p*<0.01 vs. Group HS; ^#^*p*<0.05，^##^*p*<0.01 vs. Group HS; HS, high altitude saline-treated group; HF, high altitude FJJOL-treated group; HD, high altitude diazepam-treated group; F, flatland group.

**Table S3. Exploratory behavior of mice in elevated plus maze after 3 days stimulation ($\overline{x}\pm s$).**

| **Group** | **retention time in open arm**  **(s)** | **movement distance in open arm**  **(m)** | **entries in open arm**  **(times)** | **exploratory time in open arm**  **(s)** |
| --- | --- | --- | --- | --- |
| **HS** | 34.22±38.57 | 1.97±1.25 | 1.17±0.41 | 5.87±3.97 |
| **HD** | 81.90±37.95^*^ | 6.23±2.01^**^ | 3.98±2.07^*^ | 10.18±5.28 |
| **HF** | 54.77±21.55 | 5.50±2.42^**^ | 4.67±2.80^*^ | 14.43±5.79^**^ |
| **F** | 86.44±32.16^#^ | 6.75±1.56^##^ | 4.83±1.24^#^ | 15.18±4.91^##^ |

^*^*p*<0.05, ^**^*p*<0.01 vs. Group HS; ^#^*p*<0.05, ^##^*p*<0.01 vs. Group HS; HS, high altitude saline-treated group; HF, high altitude FJJOL-treated group; HD, high altitude diazepam-treated group; F, flatland group.
